# Neoadjuvant Treatment Options in Soft Tissue Sarcomas

**DOI:** 10.3390/cancers12082061

**Published:** 2020-07-26

**Authors:** Mateusz Jacek Spałek, Katarzyna Kozak, Anna Małgorzata Czarnecka, Ewa Bartnik, Aneta Borkowska, Piotr Rutkowski

**Affiliations:** 1 Department of Soft Tissue/Bone Sarcoma and Melanoma, Maria Sklodowska-Curie National Research Institute of Oncology, 02-781 Warsaw, Poland; wiatrowka@gmail.com (K.K.); am.czarnecka@pib-nio.pl (A.M.C.); aneta.borkowska@pib-nio.pl (A.B.); piotr.rutkowski@pib-nio.pl (P.R.); 2 Department of Experimental Pharmacology, Mossakowski Medical Research Centre, Polish Academy of Sciences, 02-106 Warsaw, Poland; 3 Institute of Genetics and Biotechnology, Faculty of Biology, University of Warsaw, 02-106 Warsaw, Poland; ewambartnik@gmail.com; 4 Institute of Biochemistry and Biophysics, Polish Academy of Sciences, 02-106 Warsaw, Poland

**Keywords:** soft tissue sarcoma, neoadjuvant treatment, combined treatment

## Abstract

Due to the heterogeneity of soft tissue sarcomas (STS), the choice of the proper perioperative treatment regimen is challenging. Neoadjuvant therapy has attracted increasing attention due to several advantages, particularly in patients with locally advanced disease. The number of available neoadjuvant modalities is growing continuously. We may consider radiotherapy, chemotherapy, targeted therapy, radiosensitizers, hyperthermia, and their combinations. This review discusses possible neoadjuvant treatment options in STS with an emphasis on available evidence, indications for each treatment type, and related risks. Finally, we summarize current recommendations of the STS neoadjuvant therapy response assessment.

## 1. Introduction

Due to the growing number of available treatment options and the rarity and heterogeneity of soft tissue sarcomas (STS), the decision-making process is very complex. In comparison to surgery alone, the addition of adjuvant radiotherapy (RT) allows obtaining a high local control rate in patients with STS of extremities or trunk wall. Nevertheless, this combination does not improve patients’ overall survival (OS) [1]. Additional treatment modalities may help to improve local and distant disease control. Perioperative anthracycline-based chemotherapy (CHT) should be considered in high-risk STS [2]. However, its efficacy is still under debate. Hyperthermia (HT) may enhance the effect of both RT and CHT, but it has not been widely adopted in clinical practice [3,4,5,6]. Another novel approach includes the radiosensitization of sarcoma cells by the targeted treatment given concurrently with RT [7]. Other modalities, such as radiosensitizing nanoparticles, gave promising preliminary results, but are still under investigation in trials [8].

The introduction of neoadjuvant treatments in clinical practice was initially limited to challenging cases, such as unresectable STS, albeit currently it should be considered as an equal alternative to adjuvant therapy, bringing more advantages than risks. It is highly improbable to create a universal neoadjuvant treatment regimen for STS due to the variety of molecular subtypes and related clinical factors [9]. Thus, any clinical decision must be individualized and analyzed by STS multidisciplinary tumor board (MTB). Patients with locally advanced STS should be enrolled in clinical trials with neoadjuvant treatment; however, their availability is poor. This article summarizes the available evidence, research directions, assessment of response, and practical aspects of neoadjuvant treatment that may be helpful in the management of patients with localized STS. The options include RT, CHT, targeted therapy, HT, nanoparticles, and their combinations. Selected STS types, such as dermatofibrosarcoma protuberans, gastrointestinal stromal tumor, rhabdomyosarcoma, and Ewing sarcoma, have separate, established treatment strategies that are beyond the scope of this review.

## 2. Neoadjuvant Treatment Options

### 2.1. Radiotherapy

#### 2.1.1. Considerations and Available Evidence

Despite several advantages of neoadjuvant RT in STS and no differences in long-term outcomes as compared to adjuvant RT, neoadjuvant treatment is not widely used in clinical practice (Table 1) [10]. The main concern of neoadjuvant RT is an increase in wound complications, as described below [11]. Other important RT-related issues are delineation, RT technique, fractionation, and a postoperative boost in case of non-radical surgery.

In neoadjuvant RT, it is recommended to contour gross tumor volume (GTV) using magnetic resonance imaging (MRI) T1 post-gadolinium [12]. Clinical target volume (CTV) should be created by expanding GTV with adequate margins with additional coverage of tumor-related edema in MRI T2. In deep STS of extremities, CTV constitutes GTV +1.5-2.0 cm radially and 4 cm longitudinally. In the case of superficial STS or trunk STS, there are no clear recommendations; however, it seems reasonable to add at least 4 cm in each direction along paths of least resistance stopping at anatomical barriers. Besides regular organs at risk adequate to the anatomic site, attention must be paid to healthy skin and subcutaneous tissue, as well as the second limb, large joints, and bones. Margins in STS RT seem to be extensive. However, there is no reliable evidence to support their reduction. The only phase II Radiation Therapy Oncology Group (RTOG) 0630 non-randomized clinical trial showed that image-guided RT with concomitant margin reduction results in low late toxicity while maintaining reasonable local control in comparison to Canadian trial data [13]. Nevertheless, the numerical comparison between the RTOG-0630 and the Canadian trial is not relevant due to the difference in RT techniques. In the RTOG-0630 trial, 75% of patients were irradiated with intensity-modulated RT (IMRT), whereas in the Canadian trial in all patients older RT techniques were used [11,13].

RT techniques in STS slowly evolved from 3D-conformal RT to IMRT. IMRT allows better coverage of target volumes with the prescribed dose along with higher conformity but at the cost of an increase in volume irradiated with low doses [14]. Theoretically, irradiation of the whole extremity circumference may lead to persistent lymphedema. For this reason, many radiation oncologists have avoided using IMRT in STS. This hypothesis was not confirmed in the RTOG-0630 trial, as well as in other studies with IMRT in STS. O’Sullivan et al. conducted a phase II non-randomized prospective clinical trial with RT in STS, in which IMRT resulted in favorable functional outcomes [15]. Another analysis by Peeken et al. confirmed the good toxicity profile of tomotherapy-based IMRT in patients with STS [16].

Neoadjuvant RT in STS may be prescribed in conventional and altered fractions [17]. The conventional fractionation is 50–50.4 Gy in 25–28 fractions for 5–6 weeks; however, it is not evidence-based. Several attempts of hypofractionated RT (HFRT) were described in the literature [7,18]. The alpha/beta ratio of STS is presumably lower than 10 Gy and closer to radioresistant tumors such as prostate cancer [19,20,21]. As per the generalized linear-quadratic model, a higher dose per fraction applied to tumors with a lower alpha/beta ratio should result in better tumor control [22]. HFRT has other advantages such as shorter overall treatment time, better adherence to therapy, and higher cost-effectiveness [23,24]. However, HFRT has been validated only in phase I and phase II clinical trials in STS. Thus, it is recommended to use HFRT only in further trials or in individually selected cases upon MTB decision [7].

Unfortunately, even the best perioperative treatment cannot replace high-quality R0 surgery [25]. It has been shown that the addition of a postoperative RT boost after neoadjuvant RT and non-radical surgery does not improve local control in STS [26,27]. Nonetheless, it is still considered optional in the National Comprehensive Cancer Network (NCCN) guidelines [28].

Novel neoadjuvant RT approaches in STS include a return to abandoned spatially fractionated RT that used sieve-like collimators to produce fluctuating dose distribution with extremely high doses in selected subvolumes of a tumor. Mohiuddin et al. used spatially fractionated RT with ifosfamide-based CHT to treat bulky STS, obtaining excellent pathological responses and acceptable toxicity [29]. Dynamic RT techniques could emulate a grid-like pattern within the tumor without the use of a designed collimator. This technique is called LATTICE RT [30]. Prospective trials with LATTICE RT in locally advanced STS are awaited. Brachytherapy is not discussed in this review, because it is given intraoperatively or as an adjuvant treatment.

#### 2.1.2. Indications

According to the NCCN guidelines, neoadjuvant RT is recommended as one of the possible options in stage II, III resectable extremity, superficial trunk, or head/neck STS with acceptable functional outcomes (category 1) [28]. In the case of resectable stage II and III STS with predicted adverse functional outcomes and unresectable STS, NCCN recommends an attempt of neoadjuvant treatment, namely RT, CHT+RT, CHT, or regional limb therapy. European Society for Medical Oncology (ESMO) guidelines recommend surgery and perioperative RT as a standard treatment in high-grade (G2–3), deep, >5 cm STS [31]. The sequence of RT and surgery should be selected based on the risk of wound complications. There is no consensus regarding other clinical situations, i.e., high-grade, deep, <5 cm STS; high-grade, superficial, >5 cm STS; low-grade, deep, >5 cm STS. RT should be discussed at MTB, and the risk of local relapse, pathology, and potential toxicity should be considered.

#### 2.1.3. Risks

The most significant disadvantage of neoadjuvant RT is the increased risk of wound complications. In the Canadian trial, authors randomized patients to neoadjuvant or adjuvant RT [11]. Due to the higher occurrence of wound complications in the neoadjuvant group (35% vs. 17%), the trial was prematurely stopped. However, after a longer follow-up, more patients after adjuvant RT, than patients after neoadjuvant RT, developed fibrosis-related late RT toxicity [32]. What is important, even serious wound complications are reversible, whereas fibrosis-related late toxicities are usually permanent, progressive, and may severely deteriorate the patients’ quality of life [33]. Several patient-dependent factors increase the ratio of wound complications, such as STS localization (lower extremity and <3 mm from the skin), tumor size >10 cm, concomitant diseases as diabetes, obesity, as well as smoking [34,35,36]. Treatment-related factors that exacerbate wound complications are related to RT (as described above) and surgery (split-thickness skin graft or vascularized flap closure).

### 2.2. Chemotherapy

#### 2.2.1. Considerations and Available Evidence

The role of perioperative CHT in patients with localized STS remains controversial [37]. Neoadjuvant CHT should result in tumor burden reduction and subsequently facilitate radical surgery. In particular, CHT is expected to downstage the tumor in the case of marginally resectable STS allowing for more conservative operation [38]. Neoadjuvant CHT enables the elimination of micrometastases before massive release of cytokines induced by surgery. Finally, it should improve patient survival [39]. The neoadjuvant approach eliminates the risk of adjuvant CHT-related wound complications. Moreover, in routine clinical practice performance, the status of patients is often better before extensive surgery, enabling a more toxic but also more efficient neoadjuvant CHT regimen [40]. Better performance status indirectly allows adequate CHT dose intensity and promotes compliance. However, evidence-based confirmation of these clinical hypotheses is scarce [41].

An updated large meta-analysis of 18 randomized controlled trials of adjuvant CHT for localized resectable STS showed that anthracycline-based CHT marginally improved local recurrence, distant recurrence, overall recurrence, and OS [42]. Even less evidence justifies the use of CHT alone in a neoadjuvant setting in STS. In 2001, Eilber et al. published the results of neoadjuvant CHT of patients with locally advanced STS showing a higher percentage of pathological responses and better survival in patients receiving an ifosfamide-based regimen in comparison with patients receiving doxorubicin-based CHT (without ifosfamide) [43]. There is a lack of randomized phase III clinical trials in the field. The results of available smaller studies are contradictory [44,45,46]. In a randomized phase II clinical trial by the European Organisation for Research and Treatment of Cancer Soft Tissue Bone Sarcoma Group (EORTC-STBSG) and the National Cancer Institute of Canada Clinical Trials Group/Canadian Sarcoma Group on neoadjuvant CHT for high-risk STS, neoadjuvant CHT followed by surgery failed to show better survival than surgery alone [47]. Thus, expansion to phase III was abandoned. In the above-mentioned trial, 134 patients with resectable high-risk primary and recurrent STS were randomized to a surgery alone arm or to three cycles of doxorubicin+ifosfamide (AI) with a subsequent surgery arm. This regimen was reasonably tolerated and did not compromise surgery. Disappointingly, it resulted in unsatisfactory benefit in 5-year disease-free survival in comparison to surgery alone (56% vs. 52%) [35]. This trial was criticized for inclusion of patients with both primary and recurrent tumors as well as patients with low grade STS [48].

Histologically driven CHT showed good preliminary efficacy of selected drugs in chosen pathological STS subtypes. Four sarcoma groups (French, Spanish, Polish, and Italian) performed a phase III randomized clinical trial (NCT01710176) with neoadjuvant histologically driven CHT [49,50]. Two hundred seventy-eight patients with STS were enrolled in the trial. The patients received three cycles of epirubicin+ifosfamide (EI) or histologically driven CHT: for myxoid liposarcoma—trabectedin, for leiomyosarcoma—gemcitabine+dacarbazine, for synovial sarcoma—ifosfamide, for malignant peripheral nerve sheath tumor (MPNST)—etoposide+ifosfamide, and for pleomorphic sarcoma—gemcitabine+docetaxel. Surprisingly, after the median 52 months of follow-up, there were no differences between histologically driven CHT and EI in disease-free survival (47% in the tailored arm, 55% in EI arm, hazard ratio [HR] 1.23, 95% CI 0.88–1.73). Moreover, OS was shorter in the histologically driven arm in comparison to the EI arm (66% vs. 76%, HR 1.77, 95% CI 1.10–2.83). As a result of this trial, anthracycline-based regimens remain preferred CHT in STS regardless of histological subtype. This study also confirms that in STS patients with 60–70% risk of relapse, three cycles of neoadjuvant anthracycline+ifosfamide CHT are associated with an absolute benefit of 20% for relapse-free survival and OS.

The optimal number of CHT cycles was addressed in a study conducted by the Italian Sarcoma Group and the Spanish Sarcoma Group [51,52,53]. The researchers compared three cycles of neoadjuvant AI CHT with five cycles of the same regimen given perioperatively (three neoadjuvant cycles, surgery, two adjuvant cycles). No benefit in survival was detected between the analyzed groups. Thus, three cycles of neoadjuvant anthracycline+ifosfamide may still be considered as a preferred regimen in STS including epirubicin 120 mg/m^2^ with ifosfamide 9 g/m^2^ [42]. 

The randomized trials with neoadjuvant CHT regimens in STS are presented in Table 2.

#### 2.2.2. Indications

An advantage of neoadjuvant CHT, especially if used in combination with RT, is an improvement in the effectiveness of surgery and therefore significant improvement in patient function and postoperative quality of life. Neoadjuvant CHT may be a valuable option in patients with a low probability of OS calculated using the prognostic nomogram Sarculator [54,55]. Moreover, neoadjuvant CHT may provide benefit in patients with high-risk or marginally resectable STS [48,56,57]. NCCN suggests considering CHT as an addition to RT or as standalone neoadjuvant treatment in resectable stage II and III STS with predicted adverse functional outcomes, and in primarily unresectable STS [28]. ESMO recommends at least three cycles of neoadjuvant CHT with anthracycline+ifosfamide in selected high-risk STS patients [31]. However, the sensitivity of STS subtypes should be taken into account. The anthracycline+ifosfamide combination should be considered in more chemosensitive subtypes such as synovial sarcoma, pleomorphic sarcoma, liposarcoma and leiomyosarcoma. It is important to note that older patients (≥65 years) or those with comorbidities may not be able to tolerate such intensive treatment. More intensive CHT increases the risk of serious adverse events without providing significant benefit. Given the limited role of ifosfamide in leiomyosarcoma, doxorubicin+dacarbazine may be an alternative, less toxic treatment option in this group of patients [58]. Other treatment regimens cannot be recommended outside clinical trials. It is crucial to mention marginally sensitive and chemoresistant STS subtypes such as epithelioid sarcoma, extraskeletal myxoid chondrosarcoma, clear cell sarcoma, solitary fibrous tumor, alveolar soft part sarcoma and inflammatory myofibroblastic tumor, where neoadjuvant CHT should not be given because of lack of efficacy [59].

#### 2.2.3. Risks

In turn, a disadvantage could be a delay in surgical treatment in the case of resistance to CHT [60]. Then, in the case of tumor progression, neoadjuvant CHT should be stopped, and definitive local treatment should be performed. There is a theoretical risk of disease progression during CHT that will make surgery in primary resectable STS impossible.

Furthermore, complications of neoadjuvant CHT can considerably delay surgery and prolong overall treatment time. Toxicities depend on the used chemotherapeutic agents. Anthracyclines bring the risk of myelosuppression and cardiotoxicity, whereas ifosfamide may cause hemorrhagic cystitis, neurotoxicity, extensive vomiting, and myelosuppression [61,62,63,64,65]. Moreover, a combination of doxorubicin with ifosfamide could increase the frequency of the above-mentioned toxicities and febrile neutropenia [66].

### 2.3. Radiochemotherapy

#### 2.3.1. Considerations and Available Evidence

CHT+RT may be considered in patients with high-risk or marginally resectable STS [67]. The main aims are as follows:

Improve local control;
Symptom control, including pain relief;
Improve local response allowing for conservative surgery and negative resection margins;
Limit the metastatic spread and improve survival;
Sensitize cancer cells to RT with a possibility of RT dose reduction to avoid RT-related toxicity;
Obtain data regarding response to neoadjuvant treatment that may serve as a prognostic factor.

Guidelines favoring neoadjuvant CHT+RT are not based on high-level evidence [28,31,56,68]. The optimal sequence of treatment is unknown. CHT can be given before, after, or concomitantly with RT [55]. CHT administration before or during RT planning could serve as “stop-gap measure” giving more time for generation of complicated highly conformal RT plan [38]. Concomitant use of anthracyclines and RT brings the risk of extensive skin, mucosal, and cardiac toxicity. However, data from breast cancer studies do not confirm this hypothesis [69,70]. Studies on CHT+RT in adjuvant treatment for STS do not suggest a significant increase in toxicity, even in a subgroup of elderly patients [71,72]. In the sub-analysis of a phase III clinical trial that compared three neoadjuvant vs. three neoadjuvant and two adjuvant AI cycles, the investigator was allowed to add neoadjuvant or adjuvant conventionally fractionated RT (CFRT) to the CHT, with or without postoperative boost [53]. Neoadjuvant RT was started after the first CHT cycle and continued within the second and third CHT cycle. One hundred fifty-two patients received neoadjuvant CHT+RT. It has been shown that this combination is safe and does not have a negative impact on CHT dose intensity. The only noted significant toxicities were the slightly increased likelihood of wound complications and grade 4 thrombocytopenia.

In the literature, several attempts of the integration of neoadjuvant CHT+RT were investigated [7,18]. Various CHT regimens may be combined with both CFRT and HFRT. Such regimens are under investigation in highly locally advanced or marginally resectable STS [57]. The long-term results of novel CHT+RT regimens are presented in Table 3.

#### 2.3.2. Indications

According to NCCN guidelines, CHT+RT may be considered as a treatment option for patients with stage II, III resectable extremity, superficial trunk, or head/neck STS with acceptable functional outcomes; however, the recommendation is based upon lower-level evidence (category 2B) [28]. Furthermore, it is a treatment of choice in resectable stage II and III STS with predicted adverse functional outcomes, and primarily unresectable STS. Neoadjuvant RTH-CHTH may also provide a substantial clinical benefit allowing radical surgery in primary marginally or non-resectable STS [57].

#### 2.3.3. Risks

The addition of CHT to RT may increase toxicity. Anthracycline-based regimens may exacerbate RT mucosal and skin reactions as well as cardiac toxicity when RT is applied to the thoracic area. Moreover, even in the case of sequential CHT+RT, a phenomenon called radiation recall may occur [80]. It is defined as an inflammatory reaction in previously irradiated volume after the administration of CHT agents. Severity and length of radiation recall may vary. Finally, CHT toxicity may interrupt RT administration causing gaps in the selected fractionation regimen. Additionally, RT-induced toxicity may simultaneously decrease CHT dose intensity.

### 2.4. Targeted Therapy

#### 2.4.1. Considerations and Available Evidence

Targeted therapies inhibit specific molecules involved in the proliferation and growth of cancer cells, as well as intratumoral endothelial cells. Both vascular endothelial growth factor (VEGFR) and epidermal growth factor receptor (EGFR) are overrepresented in neoplastic cells playing an essential role in tumor angiogenesis, as well as the promotion and progression of oncogenesis and metastatic spread. Intratumoral endothelial cells are crucial in the biological effects of RT [81]. It was shown that inhibition of VEGFR and EGFR signaling pathways is an effective strategy in particular STS subtypes, and it also enhances the effect of RT in STS [82,83].

Various targeted drugs are currently investigated in metastatic STS, but also in neoadjuvant treatment, mostly with neoadjuvant RT. Despite promising efficacy, available data come from early-phase clinical trials, thus must be interpreted with caution. Moreover, some studies reported worrisome toxicity of neoadjuvant targeted therapy [7]. Further studies are necessary to use this combination in everyday clinical practice. Available reports on targeted therapies with or without neoadjuvant RT in STS are presented in Table 4.

#### 2.4.2. Indications

The combination of targeted drugs with neoadjuvant RT or targeted therapy alone should be used in prospective clinical trials or individually in selected patients [7].

#### 2.4.3. Risks

Potential toxicity of neoadjuvant targeted therapy with or without RT in STS is poorly investigated, thus unexpected early or late toxicities may occur. For example, the combination of CFRT with sunitinib or pazopanib leads to a high proportion of patients who experienced grade 3 or higher hepatotoxicity [86,92].

### 2.5. Nanoparticles

#### 2.5.1. Considerations and Available Evidence

Various radiosensitizers were tested in clinical trials in the past, mostly in head and neck cancers [93]. However, none of them is widely used in clinical practice. In recent times, in a phase II/III multicenter, international randomized clinical trial, the authors assessed the efficacy of hafnium oxide nanoparticle (NBTXR3) as a local radiosensitizer added to neoadjuvant CFRT (2 Gy to 50 Gy, 25 fractions, five weeks) [8]. The treatment was offered to patients with locally advanced resectable STS of extremities or trunk wall. In the study group patients received a single intratumoral injection of NBTXR3 before CFRT, whereas in the control group, patients received the same CFRT alone. The primary endpoint of the study was the proportion of patients with a complete pathological response.

After enrollment, randomization, and exclusion of ineligible patients, 176 patients were analyzed. Among them, 87 were in the study group and 89 in the control group. Pathological complete response was observed in 14 patients in the study group and seven patients in the control group (*p* = 0.044). Statistically significant differences were found in the proportion of patients with R0 resection, which was more frequent in the NBTXR3 group than in the CFRT-alone group. Grade 3+ CFRT-related toxicity occurred in five patients from the study group and four patients from the control group. Grade 3+ NBTXR3-related toxicity was observed in eight patients, i.e., post-injection pain in four cases and hypotension in six cases. Serious adverse events were noted in 35 patients who received NBTXR3 and 27 patients who received CFRT alone. The most frequent serious adverse event related to RT was postoperative wound complication.

To sum up, the NBTXR3 injection before neoadjuvant CFRT resulted in a higher proportion of patients with a pathologically complete response with no increase in RT-related toxicity and is a promising radioenhancer in further clinical applications. There is a lack of long-term outcomes of such treatment; thus, the real effectiveness of nanoparticles+RT in STS and the late toxicity profile are still unknown.

#### 2.5.2. Indications

In the case of approval, the nanoparticles could be used in most patients with localized advanced STS who are eligible for intratumoral injection. In the study mentioned above, patients with STS localized in the anterior abdominal region and those with a tumor volume over 3000 mL were excluded [8]. The authors explained that in the case of tumors >3000 mL, the injection of NBTXR3 would probably be unfeasible.

#### 2.5.3. Risks

The injection of nanoparticles may cause anxiety, pain, or infection within the treated site. Pain could be managed with adequate analgesia. The authors of the NBTXR3 trial reported no grade 3+ acute allergic reactions. Nevertheless, premedication with glucocorticoids should be considered before injection. There are still no data about late toxicity.

### 2.6. Hyperthermia

#### 2.6.1. Considerations and Available Evidence

The term "hyperthermia" describes a variety of methods of controlled heat delivery to cancer cells. Local or regional HT is used in conjunction with RT or CHT as a potent radio- or chemo-sensitizer [94,95]. It damages cancer cells by a direct cytotoxic effect and also provides indirect additional molecular effects, such as better tissue oxygenation, induction of apoptosis, instability of the cell membrane, dysfunction of intracellular proteins, and impairment of DNA repair [96]. Preclinical studies have confirmed this effect [97,98,99]. Routine use is limited by the low number of phase III clinical trials and poor availability of HT equipment.

In a phase III randomized multicenter clinical trial organized by the European Society for Hyperthermic Oncology and the EORTC-STBSG, the authors analyzed a group of 341 patients with localized high-risk STS [4]. The patients were randomly assigned into two arms. In the study arm, 169 patients received neoadjuvant CHT with etoposide, ifosfamide, and doxorubicin (EIA) concurrently with regional HT; in the control arm, 172 patients received EIA alone. The primary endpoint of the study was local progression-free survival. Local progression or death occurred more frequently in the control arm than in the study arm (relative hazard 0.58, 95% CI 0.41–0.83; *p* = 0.003). In the HT+EIA arm, the treatment response rate was significantly higher (28.8%) than in the EIA alone arm (12.7%, *p* = 0.002). Among patients who completed the whole treatment, OS was better in the study group (HR 0.66, 95% CI 0.45–0.98, *p* = 0.038). The main HT-related toxicities were pain, bolus pressure, and skin burn; the majority were mild or moderate.

After a median of 11.3 years of follow-up, patients from the HT+EIA arm maintained the benefit from HT [5]. In comparison to patients who received EIA alone, patients from HT+EIA had significantly better local progression-free survival (HR 0.65; 95% CI, 0.49–0.86; *p* = 0.002), survival rate (HR, 0.73; 95% CI, 0.54–0.98; *p* = 0.04), 5-year survival (62.7% vs 51.3%) and 10-year survival (52.6% vs. 42.7%).

In contrast, the effectiveness of HT+CHT in neoadjuvant treatment of locally advanced STS was proven in a phase III randomized clinical trial; evidence on HT+RT in STS treatment remains scarce. The toxicity and outcomes of neoadjuvant HT+HFRT (3.25 Gy to 32.5 Gy, ten fractions, two weeks, 4× HT) are validated in a prospective phase II clinical trial SINDIR, NCT03989596 [100].

A particularly challenging situation is reirradiation due to STS relapse or radiation-induced STS. HT might be added to enhance tumor response to RT, whose dose is limited by previous RT [101,102,103]. In a retrospective analysis, the authors assessed the outcomes of 16 patients with radiation-induced STS in the thoracic region treated with HT+HFRT using two moderately hypofractionated regimens (3 Gy to 36 Gy, 12 fractions, three weeks, 6× HT; or 4 Gy to 32 Gy, eight fractions, two weeks, 4× HT) [6]. In 13 patients, the treatment was applied with definitive intent; in three patients, it was used as adjuvant therapy to surgery. The complete response and partial response were observed in seven and two patients, respectively. The early toxicity of HT+HFRT was good. Late toxicity occurred in seven patients but was severe in only one case, i.e., ischemia of the arm on the treated side that required forearm amputation. Currently, there is one phase II clinical trial, NCT04398095, aimed at assessing the tolerance of HT+HFRT as definitive or neoadjuvant treatment for radiation-induced or recurrent previously irradiated STS [104].

What is important, HT should not be given with each fraction to avoid a reversible phenomenon called thermotolerance [105]. Cancer cells become resistant to heat-induced damage, probably due to the synthesis of heat-shock proteins and other molecular adaptation processes if heat is applied too frequently [96]. In turn, in a phase II clinical trial, the pathological response after HT+RT was better in patients who received HT twice a week than in those who received HT once a week [106].

#### 2.6.2. Indications

ESMO guidelines state that HT could be a supplementary modality to CHT [31]. Thus, HT may be offered to patients who are candidates for neoadjuvant CHT. Neoadjuvant HT+RT could be useful in patients with locally advanced STS who are not candidates for neoadjuvant systemic treatment due to poor performance status, chemoresistant pathology, or disease progression on CHT. HT+RT may also be beneficial in a selected group of patients with previously irradiated recurrent or radiation-induced STS; however, extreme caution must be taken due to the high risk of potentially severe toxicity [107]. There are no convincing data regarding optimal RT fractionation. Concurrent HT is usually applied twice a week. Treatments should be separated by at least 48 h.

#### 2.6.3. Risks

The tolerance of HT is usually excellent. Side effects are mild and include pain at the target site, blisters, skin damage, erythema, bleeding, thrombosis, infection, edema, and neuropathy [99,108]. In the case of deep regional HT, additionally, some general symptoms may occur, such as nausea, vomiting, or dyspnea. The late toxicity of HT is poorly investigated. HT should not be used for pregnant or breastfeeding women [109]. Other contraindications include the presence of metal implants or pacemakers within or in the proximity of heated tissues, unstable cardiovascular disease, epilepsy, significant neuropathy with a deteriorated sense of temperature, significant fever, or a large fluid compartment within the tumor [110].

## 3. Response Assessment

### 3.1. Radiological

A standardized approach of radiological response evaluation is crucial for a single patient who undergoes neoadjuvant treatment as well as for scientific purposes when a new regimen is assessed. Intratumoral environment variability of STS can lead to several pathological changes after neoadjuvant treatment, namely edema, hemorrhage, and necrosis, that might increase tumor volume and be wrongly misinterpreted as disease progression [75]. The analysis of 99 patients with STS treated with neoadjuvant or palliative RT has shown that in 58 patients, the tumor volume changed significantly with a volume increase in the majority of cases [111]. Moreover, a decrease in tumor size may be correlated with the presence of viable sarcoma cells, whereas "pseudoprogressing" tumors might be related to an extensive pathological response [79,112]. Thus, conventional response evaluation criteria in solid tumors (RECIST 1.1) should not be used to assess the response to neoadjuvant treatment in STS, except for myxoid liposarcomas.

EORTC-STBSG published guidelines on radiological examination and reporting after neoadjuvant RT in STS [113]. The emphasis is placed on MRI before and after RT as a suggested modality in response assessment. The article gives recommendations regarding the optimal timing and protocol of MRI. It is recommended that:

Post-RT imaging should not be performed earlier than four weeks post-RT (later if possible);

Images acquired in the same plane should be performed with identical planing and slice thickness to allow correlation between sequences;

Except for myxoid liposarcomas, size and volume measurements should not be used to reflect histopathological response;

Internal signal/density characteristics should be used in combination to assess response;

Areas of new enhancement should be interpreted with caution as they can arise secondarily to vascular disruption following RT and do not necessarily reflect progression;

Not all areas of diminished enhancement following RT represent necrosis and, therefore, attention to terminology is suggested. The term “treatment effects” may be more appropriate encompassing several processes, such as necrosis, cystic change (liquefaction), or hyalinization.

Special attention was paid to functional imaging. The authors gave recommendations regarding parameters for reporting multiparametric MRI in clinical trials.

### 3.2. Pathological

Treatment-induced tissue necrosis is a predictive factor of patient survival in bone sarcomas. Several attempts failed to show this dependency in STS [114,115]. At the same time, meta-analysis of 21 studies comprising 1663 patients has confirmed that tumor necrosis <90% following neoadjuvant therapy in STS is associated with increased recurrence risk and shorter OS [116].

For pathology reports, several pathological factors were considered in various studies until the proposal of a standardized pathological examination and reporting of STS resection material after neoadjuvant treatment was published by EORTC-STBSG [117]. The article describes the process of specimen documentation. The most important innovation is the introduction of the five-grade scale of microscopic evaluation based on the proportion of tumor area that is viable. The assessment is based on "stainability," which means the visualization of nuclei by hematoxylin. The percentage of stainable cells should represent the whole specimen; thus, the representative slab should be supplemented with additional blocks in the final response score. The categories include:

A: no stainable tumor cells;
B: single stainable tumor cells or small clusters (overall below 1% of the whole specimen);
C: >=1%–<10% stainable tumor cells;
D: >=10%–<50% stainable tumor cells;
E: >=50% stainable tumor cells.

The authors were not able to give any recommendations regarding immunohistochemical markers that may be useful in the assessment of pathological response.

### 3.3. Biomarkers

There are as of yet no specific biomarkers that would predict patient survival or, even better, indicate the optimal type of therapy. The general importance of using proper markers has been pointed out by Mortaji and Lebduska [118]. Kondo and Kawai suggested investigating (for STS) numerous potentially useful molecular proteomic markers which have contributed to cancer therapy, but they did not follow up on their suggestions [119]. However, in 2019 Burns et al. stated that no proteomic markers for STS had reached the clinic [120]. Kane et al. examined specimens from 60 STS patients, but pretreatment samples were available for only 23 and matched samples pre- and post-treatment for 12 [121]. They analyzed the expression of several genes involved in cell-cycle regulation and hypoxia but did not find (possibly because of the small size of the group) any association of pretreatment expression of any of the markers with survival.

Schenone and Van Tine have identified seven biomarkers potentially useful for therapies commonly used in STS treatment (except for gastrointestinal stromal tumors) [122]. These are TOP2A (topoisomerase IIA for anthracycline), RRM1 (ribonucleotide reductase M1 unit for gemcitabine and Taxotere), TLE3 (transducing-like enhancer protein 3 for taxanes), MGMT (O6-methylguanine-DNA-transferase for temozolomide and dacarbazine), TUBB3 (tubulin beta-3 chain for taxanes and vinca alkaloids), SPARC (secreted protein acidic and rich in cysteine for taxanes) and PTEN (phosphatidylinositol-3,4,5-triphosphate 3-phosphatase for mTOR inhibitors), and the authors have carefully analyzed earlier studies; however, most of them did not concern STS.

More recently, Caruso and Garofalo analyzed pharmacogenomic biomarkers for STS therapies [123]. These biomarkers would be potentially useful in predicting drug responses in patients. They have analyzed the literature for germline and somatic biomarkers and suggest that next-generation sequencing technologies and larger gene panels would be useful in obtaining results that could be implemented in the clinic.

In the clinical trial NCT01710176, Pasquali et al. used a 67-gene expression-based signature to stratify 87 patients into lower-risk and higher-risk groups; however, no differences were observed between them in disease-free and OS, even though this set of markers, CINSARC (complexity index in sarcomas) had been previously tested in retrospective studies [124]. Other STS biomarker clinical studies are ongoing.

## 4. Novel Approaches and Future Directions

### 4.1. Molecular Targeted Therapy

The only routinely used highly effective neoadjuvant subtype-targeted therapy in STS is imatinib for unresectable localized dermatofibrosarcoma protuberans and gastrointestinal stromal tumors [125,126]. However, being a diversified and orphan group of tumors, STS present various genetic alterations that may be potential targets for novel targeted therapies. Possible genetic pathways include specific translocations (for example, anaplastic lymphoma kinase [*ALK]* fusion in inflammatory myofibroblastic tumors), gene amplifications (for example, the amplification of *MYC* in radiation-induced angiosarcomas), oncogenic mutations (for example, activating mutations in the *KIT* receptor), and complex genomic rearrangements (vast majority of STS) [127]. A clear example is an inhibitor of enhancer of zeste homolog 2 (*EZH2)* histone methyltransferase, tazemetostat, registered in the USA for metastatic or locally advanced unresectable epithelioid sarcoma [128]. A mutation in the *INI1* suppressor gene in epithelioid sarcoma cells causes deregulation of *EZH2* that leads to the activation of multiple oncogenic signaling pathways. Tazemetostat competitively inhibits *EZH2,* stopping epithelioid sarcoma growth. Another effective novel therapy, namely the inhibitor of tropomyosin receptor kinases A, B and C, targets *NTRK* fusions. An analysis of databases from three ongoing phase I or II clinical trials with entrectinib (ALKA-372-001, STARTRK-1, and STARTRK-2), which enrolled 54 patients with metastatic or locally advanced NTRK fusion-positive solid tumors, showed a high ratio of objective durable responses (31/54 patients, median duration 10 months) with good treatment tolerance [129]. In the study, STS were the predominant group of tumors (13 patients, 24%). The objective response was observed in almost half of them (6/13 patients, 46%). Several other subtype-targeted molecules are currently under investigation [127,130,131].

### 4.2. Immunotherapy

Promising results of immune checkpoint inhibitors in various cancers encouraged investigators to assess their potential in STS. This group of molecules targets the most important regulators of the immune system that are responsible for anticancer response. Immune checkpoint inhibitors are especially active in tumors with a high mutational burden. Approved molecules include anti-CTLA4, anti-programmed cell death 1 (PD-1), and anti-PD-1 ligand immunotherapies. Despite a strong theoretical basis and expectations, preliminary results from phase I and II clinical trials showed moderate activity of immunotherapy in STS [132]. The exception is alveolar soft part tissue sarcoma, a rare and radiochemoresistant STS subtype. In a phase II clinical trial with axitinib and pembrolizumab in patients with advanced alveolar soft part tissue sarcomas and other STS subtypes, the 6- and 12-months progression-free survivals were 47% and 28%, respectively [133]. The best overall response rate was described in eight patients and, among them, six had alveolar soft part tissue sarcoma. Another report described similar activity of immunotherapy in this rare STS subtype [134]. Nevertheless, immunotherapy has not been investigated in nonmetastatic or resectable STS. Thus, it should not be recommended as a neoadjuvant treatment. New clinical trials are required to assess the efficacy of immunotherapy in STS.

## 5. Practical Recommendations and Conclusions

### 5.1. Particular Clinical Situations

#### 5.1.1. High Risk Soft Tissue Sarcomas

Patients with high-risk STS may benefit from neoadjuvant therapy. Neoadjuvant RT should be a part of the treatment provided that a risk of wound complications is acceptable. Due to high risk of distant metastases, RT may be combined with neoadjuvant CHT. Anthracycline–ifosfamide CHT regimens are preferred regardless of STS subtype. In fragile patients, less intensive CHT regimens, such as anthracyclines alone, may provide adequate efficacy without the risk of a significant increase in CHT toxicity.

#### 5.1.2. Locally Advanced Low-Grade Soft Tissue Sarcomas

Slowly growing locally advanced low-grade tumors bring a low risk of metastatic spread. Thus, administration of CHT is not recommended. This group of STS is relatively more radioresistant. Therefore, based on radiobiological models, hypofractionated RT might provide benefit in local response. Additional radiosensitizing modalities such as HT may enhance efficacy of RT.

#### 5.1.3. Marginally Resectable or Non-Resectable Soft Tissue Sarcomas

Neoadjuvant therapy is the gold standard for the treatment of marginally resectable or non-resectable STS. Both CRFT and HFRT may be considered. In bulky, symptomatic tumors, shorter RT regimens are preferred. RT might be combined with anthracycline-based CHT to increase local response and decrease the risk of distant metastases. Due to the usually large volume of those tumors and related symptoms, the application of regional HT or nanoparticles may be problematic or even unfeasible.

#### 5.1.4. Chemoresistant Sarcoma Subtypes

Epithelioid sarcoma, extraskeletal myxoid chondrosarcoma, clear cell sarcoma, solitary fibrous tumor, alveolar soft part sarcoma and inflammatory myofibroblastic tumor constitute STS subtypes resistant to conventional cytotoxic chemotherapy. In those subtypes, neoadjuvant CHT is not recommended due to lack of efficacy and risk of disease progression. In the case of resectable chemoresistant STS, the preferred neoadjuvant approach is RT followed by surgery. More locally advanced and marginally resectable chemoresistant STS may benefit from additional radiosensitizing modalities, such as HT or nanoparticles. Although no targeted therapies have been investigated as a neoadjuvant treatment specifically in chemoresistant STS, tyrosine kinase inhibitors and antiangiogenic agents may be considered individually in selected cases. The only exception is the inflammatory myofibroblastic tumor presenting the ALK gene mutation, which is susceptible to ALK inhibitors [135].

#### 5.1.5. Radiation-Induced or In-Field Recurrent Soft Tissue Sarcomas

The management of radiation-induced or in-field recurrent STS is challenging. The only curative modality in localized tumors is radical resection with wide negative margins. The role of secondary RT is unclear, mostly due to the concerns about possible severe side effects after re-irradiation. However, RT may be carefully considered in selected cases, especially after a long period from the first RT, and absence of significant late toxicity from previous irradiation. HT may allow RT dose reduction without a decrease in treatment efficacy. CHT and targeted therapy may be used in metastatic disease, but their role in neoadjuvant therapy is not established.

### 5.2. Conclusions

There are multiple options for neoadjuvant treatment in STS that are focused on improving local and distant control. Any neoadjuvant approach should be considered individually at the MTB, taking into consideration tumor site, stage, pathology, comorbidities, age, resectability, institutional protocols, and availability of methods. The authors’ consensus on available combinations that could be considered in various clinical situations is presented in Table 5. The response to treatment should be assessed by standardized radiological and pathological criteria. New clinical trials with new combinations of methods in the neoadjuvant setting are encouraged.

## Figures and Tables

**Table 1:** Comparison of neoadjuvant and adjuvant radiotherapy in soft tissue sarcomas.

Issue	Adjuvant Radiotherapy	Neoadjuvant Radiotherapy
Delineation	Complicated (no GTV, fusion with preoperative imaging, postoperative changes)	Easy (visible GTV)
Target volume	Larger (tumor bed, scars, drainage, operative route, and margins)	Smaller (GTV + margin)
Healthy tissues	Move to the tumor bed	Pushed away by the tumor
Dose	Higher (60–66 Gy EQD2)	Lower (45–50.4 Gy EQD2)
Treatment time	Longer	Shorter
Hypofractionation	No/not known	Possible
Pathological assessment	Unhindered	Hindered
Tumor response	None	Possible
Resection margins	No influence	Could improve
Tumor seeding during resection	No influence	Possible reduction
Risk of early toxicity ^1^	Lower	Higher
Risk of late toxicity ^1^	Higher	Lower
Combination with chemotherapy	Possible	Possible

^1^ In conventionally fractionated radiotherapy; abbreviations: EQD2—equivalent total dose in 2-Gy fractions; GTV—gross tumor volume.

**Table 2:** Neoadjuvant chemotherapy in soft tissue sarcomas.

Authors and Type of Study	N	Treatment Arms	Perioperative Radiotherapy	Response Rate	Disease-Free Survival @Years	Overall Survival @Years
Gortzak et al. 2001 phase II randomized trial [47]	67	3 × AI + surgery	46%	28%	56% @5y	65% @5y
	67	surgery	54%	-	52% @5y	64% @5y
Gronchi et al. 2012, 2016 phase III randomized trial [51,52]	160	3 × EI + surgery	97%	23%	56% @10y	64% @10y
	161	3 × EI + surgery + 2 × EI	93%	19%	58% @10y	59% @10y
Gronchi et al. 2017, 2019 phase III randomized trial [49,50]	145	3 × EI + surgery	79%	14%	55% @5y	76% @5y
	142	histologically driven CHT + surgery	80%	6%	47% @5y	66% @5y

Abbreviations: AI—doxorubicin, ifosfamide; CHT—chemotherapy; EI—epirubicin, ifosfamide; N—number of patients; NR—not reported.

**Table 3:** Novel neoadjuvant radiochemotherapy regimens in soft tissue sarcomas.

Authors and Type of Study	N	Radiotherapy Regimen	Chemotherapy Regimen	Hematology Toxicity	Local Control @Years	Overall Survival @Years
Temple et al. 1997 prospective cohort [73]	42	10 × 3 Gy	Concurrent ADM	not reported	97% @5y	65% @5y
Edmonson et al. 2002 phase II clinical trial [74]	39	25 × 1.8 Gy	Concurrent IMAP	G3+ 77%	92% @5y	80% @5y
DeLaney et al. 2003 phase II clinical trial [75]	48	22 × 2 Gy	Interdigitated MAID	febrile neutropenia 25%	92% @5y	84% @5y
Kraybill et al. 2006 phase II clinical trial [76]	64	22 × 2 Gy	Interdigitated MAID	G4 83%	90% @3y	75% @3y
Ryan et al. 2008 phase II clinical trial [77]	25	8 × 3.5 Gy	Concurrent EPI+IFO	G4 84%	88% @2y	84% @2y
MacDermed et al. 2010 retrospective cohort [78]	34	8 × 3.5 Gy	Concurrent IFO	G4 53%	89% @5y	45% @5y
Hong et al. 2013 retrospective cohort [79]	66	22 × 2 Gy	Interdigitated MAID	febrile neutropenia 10%	91% @5y	86% @5y
Spalek et al. 2019 phase II clinical trial [57]	30	5 × 5 Gy	Interdigitated AI	G3+ ^1^ @26%	97% @1y	not reported

^1^ Only grade 3 or higher toxicities that caused chemotherapy dose reduction or withdrawal; abbreviations: ADM—doxorubicin; AI—doxorubicin, ifosfamide; EPI—epirubicin; FN—febrile neutropenia; G—grade; IFO—ifosfamide; IMAP—ifosfamide, mitomycin, doxorubicin, cisplatin; MAID—mesna, doxorubicin, ifosfamide, dacarbazine; N—number of patients.

**Table 4:** Neoadjuvant targeted therapies in soft tissue sarcomas.

Type of Trial	Treatment Regimen	N	Results	Toxicity
phase II non-randomized [84]	4 doses of bevacizumab (dose every 2w) + RT 28 × 1.8 Gy (5.5w)	20	≥80% necrosis in 45%; pCR in 15%	G3+ 20%; wound complications 20%
phase II randomized NCT01446809 [85]	AI + placebo (2w) vs AI + pazopanib (2w) and optional RT	21	no differences in maximal SUV	SAE: pazopanib 64%; placebo 57%
phase I non-randomized PASART-1 [86]	pazopanib 400, 600 or 800 mg daily (6w) + RT 25 × 2 Gy (5w)	12	≥80% necrosis in >70%; near pCR in 40%	G3+ hepatotoxicity 27%; wound complications 20%
phase II non-randomized NOPASS [87]	pazopanib 800 mg daily (3w)	21	≥50% reduction in SUV only in one case	G3 33.3%; G4 ^1^ 4.8%
phase II non-randomized PASART-2 [88]	pazopanib 800 mg daily (3w) + RT 18 × 2 Gy (3.5 w)	not reported	terminated	not reported
phase I non-randomized NCT00864032 [89]	sorafenib 2x200, or 200/400 mg daily + RT 25 × 2 Gy (5w)	8	pCR in 38%	G3+ 50% (in 200/400 mg arm only); severe wound complications 38%
phase I non-randomized [90]	sorafenib 200, 400, or 2x400 mg daily started 2w before chemotherapy + 3 × EI + RT 8 × 3.5 Gy (1.5w)	16	pCR in 44%	G4 hematological 88%; severe wound complications 38%
phase II non-randomized SunRaSe [91]	sunitinib 25 mg or 37.5 mg daily (7.5w) + RT 28 × 1.8 Gy (5.5w)	9	pCR in 33%	G3+ 67%; severe wound complications 22%
phase Ib/II non-randomized SUNXRT [92]	sunitinib doses 37.5-50 mg (7.5w) + RT 28 × 1.8 Gy (5.5w)	9	terminated (toxicity); necrosis in 75%	G3+ 78%; G3+ hepatotoxicity 44%; G3+ late toxicity 22%

^1^ Anastomotic leakage; abbreviations: AI—doxorubicin, ifosfamide; CHT—chemotherapy; EI—epirubicin, ifosfamide; G—grade; pCR—pathological complete response; RT—radiotherapy; SAE—serious adverse event; SUV—standardized uptake value; w—weeks.

**Table 5:** Combinations of neoadjuvant treatment in soft tissue sarcomas in various clinical situations: authors’ consensus.

Clinical Situation with Localized Soft Tissue Sarcoma	Recommended Neoadjuvant Therapy	Methods not Recommended
High risk	Radiotherapy	-
	Chemotherapy	
	Radiotherapy + chemotherapy	
	Chemotherapy + hyperthermia	
Locally advanced low-grade	Radiotherapy ^1^	Chemotherapy
	Radiotherapy ^1^ + hyperthermia ^2^	
Marginally resectable and non-resectable	Radiotherapy ^1^ + chemotherapy	Hyperthermia ^3^
	Radiotherapy ^1^	
Chemoresistant subtypes	Radiotherapy	Chemotherapy
	Radiotherapy + hyperthermia ^2^	
	Radiotherapy + targeted therapy ^2^	
	Targeted therapy ^2,4^	
Radiation induced or in-field recurrent	Chemotherapy	Radiotherapy in case of early recurrence or significant late toxicity
	Chemotherapy + hyperthermia	
	Radiotherapy + hyperthermia ^2^	
	Radiotherapy	

^1^ Hypofractionated regimens should be considered; ^2^ Experimental (clinical trials); ^3^ unfeasible in most cases; ^4^ routinely only in dermatofibrosarcoma protuberans.
